# Designing a filler material to reduce dielectric loss in epoxy-based substrates for high-frequency applications†

**DOI:** 10.1039/d4ra07419j

**Published:** 2025-01-09

**Authors:** Ilkan Calisir, Elliot L. Bennett, Xiantao Yang, Jianliang Xiao, Yi Huang

**Affiliations:** a Department of Chemistry, University of Liverpool Grove Street Liverpool L69 7ZD UK jxiao@liverpool.ac.uk; b Department of Electrical Engineering and Electronics, University of Liverpool Brownlow Hill Liverpool L69 3GJ UK huangyi@liverpool.ac.uk

## Abstract

In response to the demand for epoxy-based dielectric substrates with low dielectric loss in high-frequency and high-speed signal transmission applications, this study presents a surface-engineered filler material. Utilizing ball-milling, surface-modified aluminum flakes containing organic (stearic acid) and inorganic (aluminum oxide) coatings are developed. Incorporation of the filler into the epoxy matrix results in a significant increase in dielectric permittivity, *ε*_r,_ by nearly 5 times (from 4.3 to 21.2) and nearly an order of magnitude reduction in dielectric loss, tan *δ*, (from 0.037 to 0.005) across the 1 to 10 GHz frequency range. Extension of this method to glass fabric-reinforced epoxy-based substrates, resembling widely used FR4 in printed circuit boards, exhibits minimal permittivity variation (4.5–5.4) and considerable reductions in dielectric loss (from 0.04 to 0.01) within the same frequency range. These enhancements are attributed to improved filler dispersion and suppression of electron transport facilitated by double-layer coatings on the flake surface under varying electric fields. The findings highlight the potential of surface-modified aluminum flakes as a promising filler material for high-frequency and high-speed substrate applications requiring low-loss.

## Abstract 2

We present a new surface-engineered filler material—stearic acid-coated aluminum flakes—designed for use in epoxy-based dielectric substrates to significantly reduce dielectric loss.

## Graphical abstract

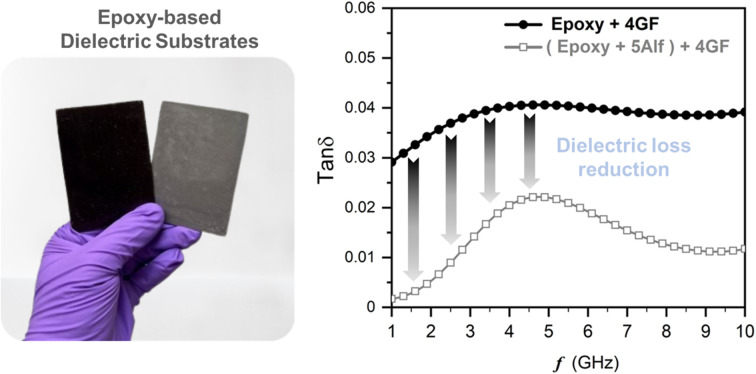

## 1. Introduction

With the advancement of information and communication technology, notably the advent of fifth-generation (5G) communication technology, there is a notable trajectory towards the development of electronic devices and equipment geared for high-frequency and high-speed signal transmission. This evolution encompasses a range of products, from consumer electronic terminal products to communication base stations.^1,2^ This trend underscores greater demand for advanced substrate materials that meet increasingly stringent requirements, particularly in terms of ensuring low dielectric loss (*D*_f_ or tan *δ*) while achieving the requisite dielectric constant (*D*_k_ or *ε*_r_) values depending on the specific application context. Conversely, the rise in operating frequency and power, alongside enhanced integration in electronic devices, inevitably leads to increased losses, mainly associated with dielectric materials and conductors, as well as greater residual heat generation.^3^ Consequently, there is a high demand in dielectric substrate materials offering low tan *δ* with improved thermal management capabilities.

In general, the ideal dielectric substrate material should possess high thermal conductivity, low dielectric constant, and minimal dielectric loss. This combination allows for effective heat dissipation, preventing signal transmission delays and reducing energy loss.^1,3^ Effectively dissipating heat while not affecting the dielectric properties becomes a significant challenge, particularly in devices operating at high frequencies (GHz). Therefore, materials that are both thermally conductive and electrically insulating are becoming increasingly crucial for enhancing device reliability. Polymers as insulating materials, including polytetrafluoroethylene (PTFE), polystyrene (PS), polyethylene (PE), polyimide (PI), polypropylene (PP), silicone elastomers and epoxy resin-type polymers, *etc.*, have found widespread use in the electrical and electronics industries due to their typically low dielectric loss (ranging from 10^−2^ to 10^−4^) and varying dielectric constant (ranging from 2 to 10).^4–7^ Among them, PTFE, liquid crystal polymer (LCP), and PI have gained extensive usage as circuit board substrates.^3,6^ Additionally, epoxy resin-type substrate materials, renowned for their excellent mechanical strength and adhesion properties to metal components, stand out as the predominant choice for copper-clad laminates used as printed circuit boards (PCBs).^8–12^ This preference is attributed to their cost-effectiveness, and versatility in serving as a polymer matrix in a woven glass fabric reinforced composite. These epoxy resin-type materials exhibit moderate dielectric properties, with a dielectric constant typically ranging between 3 and 5 and a relatively low dielectric loss tangent of 0.02.^3,9,11,12^

**Fig. 1:**
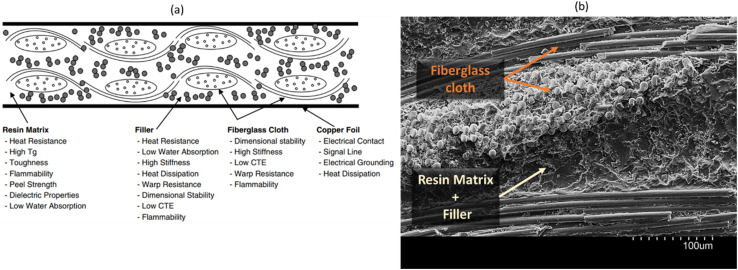
(a) A schematic illustrating the layout of a typical FR4 substrate, detailing the components and their respective roles;^15^ (b) scanning electron microscopy (SEM) image of cross-sectioned FR4 substrate.

PCBs, FR4 as a common example, play a crucial role as substrate materials in the electronic module market, serving as the foundation for mounting electronic chips and forming circuit modules.^1,13^ FR4 comprises copper foil and insulating materials, and the insulator part is a laminate composite comprised of an epoxy resin matrix, several layers of woven fiberglass cloth and inorganic fillers. The roles of each component within the laminate are given in Fig. 1a. FR4 substrate is cross-sectioned, and its SEM image is shown in Fig. 1b. Materials for PCB require various properties including thermal stability, mechanical strength, moisture resistance, flame retardancy and electrical insulating performance. As the use of electronic modules expands into the microwave-frequency range with rising signal rates, achieving low-loss in FR4, in a cost-effective manner, becomes a paramount requirement. However, the resin, a fundamental component of FR4, generally exhibits high-loss properties, primarily due to its main constituent, epoxy.^11,14^ Although the dielectric property of the filler material is anticipated to play a significant role in FR4 composite, it has been relatively underexplored compared to the dominant influence of the resin. Notably, materials research in PCBs has been actively pursued across various areas,^15^ including enhancing their thermal management capabilities,^1,12^ exploring sustainability/recyclability,^16–18^ improving mechanical/adhesion properties,^10,14^ investigating the utilization of natural fibers as reinforcement,^14,19^ and their electrical/dielectric properties.^11^ Among these areas, dielectric properties, particularly the dielectric loss (*D*_f_, dissipation factor), are regarded as the most critical factor in PCB applications. As electronic devices' operating frequencies rise to higher bands and electronic components shrink in size, this trend can potentially lead to signal delays and higher energy losses within electronic components at high frequencies.

Adding nano or micro sized inorganic fillers into the polymer matrix to form polymer composites are common practice for tuning the dielectric properties. These fillers can directly be used as-synthesized or can undergo post processing after synthesis to achieve the desired dielectric properties. For instance, high permittivity ceramics (BaTiO_3_, CaCu_3_Ti_4_O_12_, and MXeNe),^20–23^ (semi)conductive particles (carbon, MoS_2_, Zn, Al, Ni, Ag)^24–32^ in various shapes and sizes, or a combination of these fillers^33,34^ are widely studied and modified with the aim to enhance the interfacial polarization and thereby increase the permittivity of polymer composites. On the other hand, the strategies for lowering or suppression dielectric loss of a polymer material/composite can be achieved *via* (i) intrinsic molecular design including introducing groups with low polarizability or bulky pendant groups to the polymer structures, reducing the number of polar groups, fluorination, or minimizing intermolecular friction;^5,6,12,35–37^ (ii) introducing charge traps, pores(foaming) or nano–micro sized low-loss inorganic fillers into the polymer matrix to form polymer composites, such as low permittivity and low loss ceramics (SiO_2_ and Al_2_O_3_);^4,5,38,39^ (iii) surface modification of inorganic fillers (*e.g.* decorating/depositing nano-sized metals, coating with certain polymers) used in a dielectric host.^40–45^ Multilayering technology, which laminates different layers with varying permittivity/dielectric loss, has also been recently found to be an effective strategy to improve the dielectric performance of a polymer composite.^29,46^

In this work, we developed a filler material to reduce the dielectric loss of the epoxy host. The selected filler material is self-passivated aluminum metal, whose surface is modified through ball-milling in the presence of stearic acid. Due to its high malleability, the shape of the resulting metal particles after the ball-milling operation is transformed from irregular to flake-like, which can then be added to the epoxy resin. Generally, the addition of conductive metal fillers to the polymer matrix results in the formation of a micro-capacitive grid with the filler acting as the electrode and the polymer as the dielectric, which may cause the composite to approach the ‘percolation threshold’, where conductive pathways are increasingly formed, leading to initially monotonic then a sharp increase in both the real and imaginary parts of permittivity, and the resulting composites can exhibit very high loss (tan *δ* > 0.1) even at fractions as low as 0.5 wt%.^29^ This phenomenon is commonly reported in many such percolative systems.^25,26,47–49^ With Al metal particles-loaded epoxy composite, reaching percolation is found to be much greater (up to 85 wt% of metal loading) than most metals, which is attributed to the robust and high insulating character of self-passivated oxide layer formed on the Al core^28^ and the oxide layer thickness on Al flake surface can be increased through thermal oxidation.^31^ Recent work of Cao *et al.* showed that the high permittivity and low loss in Al-loaded epoxy composites can be achieved *via* application of uniaxial pressure during processing, leading to a relatively lower percolation threshold and effectively increasing microcapacitor formation through ultrathin Al_2_O_3_ layer on the spherical Al particles.^50^ However, due to the insulator–conductor transition at the percolation threshold, the dielectric loss of these percolative composites also dramatically increases in the vicinity of the percolation threshold, which counteracts the benefits of the enhancement in the dielectric constants. Recently, some attempts have been made to reduce the dielectric loss by introducing interlayers or shells between the conductive fillers;^28,33,34,47,48^ thus, the fillers can be prevented from contacting with each other directly. Nevertheless, these efforts typically succeed in either suppressing or maintaining the dielectric loss of the polymer matrix at the same level but often fall short of achieving a significant reduction in the loss, and the research on finding potential filler materials to reduce the dielectric loss has received limited attention, with common low-permittivity, low-loss ceramic fillers such as silicon dioxide (SiO_2_ or silica) and silicates being the predominant choice for FR4 composites in both nanoparticles or glass fiber forms.^15,42,51^

Surface modification processes to customize the interfaces between the matrix and the filler have been extensively studied in the past decade to enhance dielectric performance. These modifications can enhance the bonding between a filler and host, and affect the overall polarizability of the polymer/ceramic matrix by enhancing the hydrophobicity and uniformly dispersing the fillers,^52,53^ resulting in better-performing insulating materials, for instance using silane coupling agents^40^ and fatty acids^41,54^ in ceramics and metals as dispersion and process control agents, respectively. Building on this concept and applying it to a self-passivated conductive filler is the driving force behind this research. It has been found that employing the developed filler in this manner can greatly reduce the dielectric loss of the epoxy matrix.

Specifically, we developed flake-shaped aluminum particles through ball-milling, incorporating well-insulated organic–inorganic protective layers. This selection was motivated by their ability to establish micro–nano capacitor networks within polymer matrices, while effectively reducing interfacial polarization loss in the composite due to surface coating. Such filler material is found to be highly effective at reducing the dielectric loss of a host material when it is added. More specifically, we focused on epoxy and glass fabric-reinforced epoxy-based substrates for demonstration purposes. This study aims to investigate the impact of a new filler, *i.e.* flake-shaped surface-modified aluminum metal flakes (Alf), on the dielectric loss properties of epoxy and glass-reinforced epoxy substrates, commonly used in PCBs.

## 2. Results and discussion

**Fig. 2:**
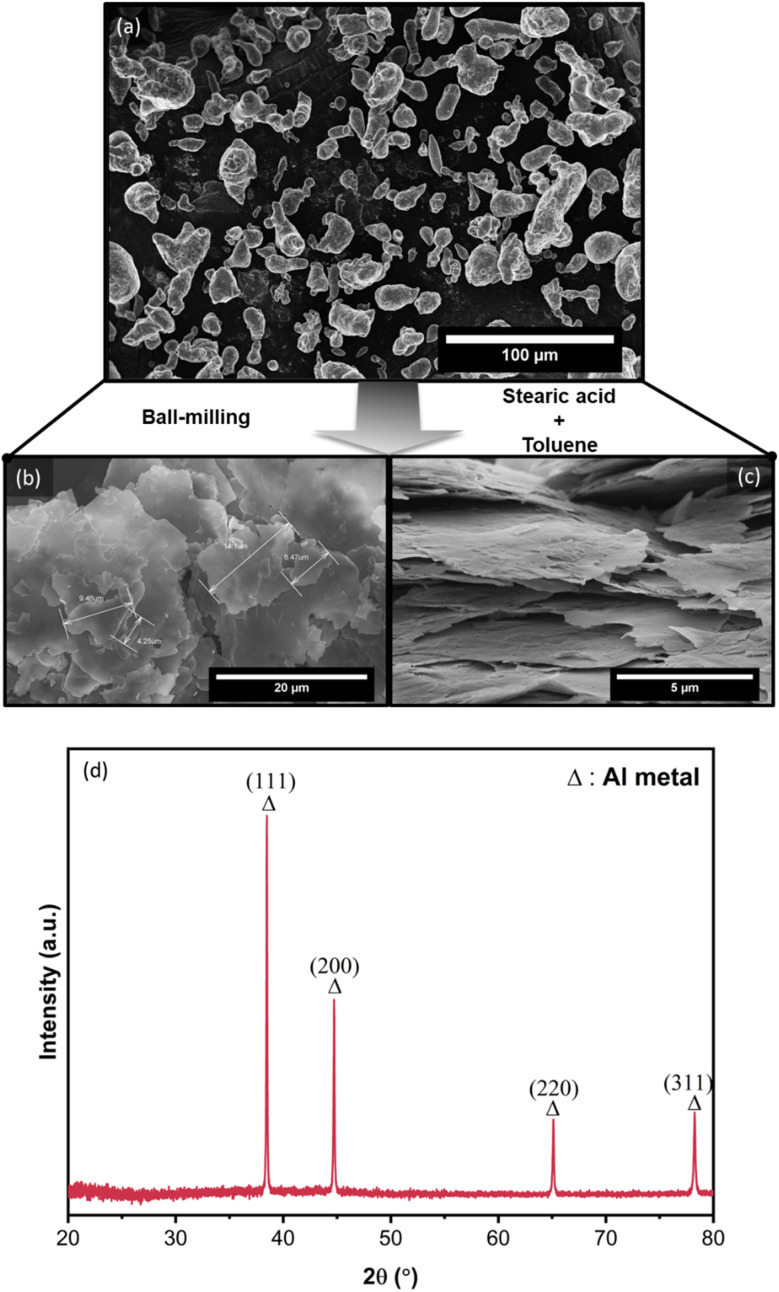
SEM images of (a) as-received Al powder; (b–c) obtained stearic acid-coated Alf by the ball-milling of Al powder. (d) XRD profile of resulting Al flakes (Alf).

Alf particles were obtained by the ball-milling of Al powder with the presence of a processing control agent, stearic acid (C_18_H_36_O_2_, a fatty acid), and toluene as a non-reactive solvent/lubricant. The transformation from irregularly shaped Al particles to Al flakes can be seen *via* SEM images as shown in Fig. 2a–c. The size of the flakes varies in the range of 1 to 15 μm in lateral and 50–100 nm in thickness. Due to their small size and shape, the flakes are prone to aggregation and are found to be stacked, forming agglomerates in 30 to 100 μm size. Fig. 2d shows the XRD profile of obtained Al flakes, which exhibits the characteristic diffraction peaks of Al metal associated with the typical structure of cubic Al (ICSD#18839).^55^ Aluminum is widely known as a self-passivating metal, wherein exposure to air or oxygen leads to the formation of a self-passivated amorphous aluminum oxide layer, serving as an insulating boundary layer outside the metallic core. During the production of flakes by ball-milling, an additional organic coating layer, stearic acid, is attached to this self-passivated inorganic layer. As these coating layers (organic–inorganic layers) are primarily amorphous, X-ray diffraction (XRD) analysis typically did not detect any crystalline phases beyond the Al metal phase within its detection limits.

The organic coating agent, stearic acid, is selected for a number of reasons:^54,56–60^ (i) as a processing control agent to prevent cold-welding of aluminum metal particles during ball-milling; (ii) promoting effective flake formations by inhibiting the over-oxidation of milled surface of aluminum particles; (iii) providing hydrophobicity to the flake particles, increasing the dispersive properties; (iv) providing an additional electrically insulating layer on the flake surface thanks to non-polar hydrophobic tails; and (v) being a cost-effective, chemically robust, safe-to-handle and non-hazardous natural material. Various types of organic coatings, including behenic acid, lauric acid, perfluorodecanoic acid, and propyltrimethoxysilane, were also utilized for coating Al flakes. However, stearic acid was found to perform the best in terms of effective flake formation and boosting dielectric permittivity with significant loss reduction. All optimization and screening steps of the organic coatings are detailed in the ESI, Fig. S1 to S8.† As a control experiment, aluminum flakes were produced without the addition of stearic acid. For safety reasons, this process was conducted in a polypropylene jar filled with nitrogen using a low-energy benchtop roller mill. It was observed that the flakes partially underwent cold welding and showed a lack of hydrophobicity (Fig. S12d to f†). Additionally, achieving a comparable flake size to those produced with stearic acid in high-energy ball milling required over 192 hours. When these uncoated flakes were incorporated into an epoxy matrix, they exhibited relatively higher dielectric losses compared to stearic acid-coated Al flakes (Fig. S12g†).

**Fig. 3:**
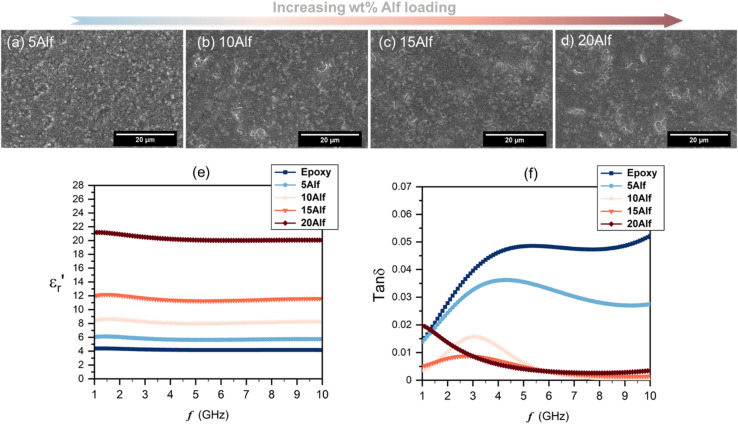
SEM images of the surface of Alf-added epoxy composites with varying wt% of Alf; (a–d) 5, 10, 15 and 20, labelled as 5Alf, 10Alf, 15Alf and 20Alf, respectively (scale bar is 20 μm). Frequency-dependent dielectric properties of epoxy and Alf-added epoxy composites; (e) the real part of permittivity, 
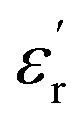
, and (f) dielectric loss, tan *δ*, with various wt% Alf.

SEM images of the composites are shown in Fig. 3a–d. In the images, the flakes within the epoxy host are distinguishable due to their bright white features, outlining the edges of the filler, while the darker gray regions predominantly represent the epoxy matrix. Up to a loading of 15 wt% (15Alf), the aluminum flakes are dispersed relatively evenly within the epoxy matrix, with no significant agglomeration observed. However, in the case of the 20Alf composite, there is a slight agglomeration of flakes indicating inadequate dispersion, requiring additional dispersion steps.

The frequency-dependent dielectric properties of both epoxy and Alf-loaded epoxy composites are assessed through the DAKTL-2 system utilizing an open coaxial probe across the frequency range from 1 to 10 GHz at room temperature. The measured dielectric properties, including the real part of complex permittivity 
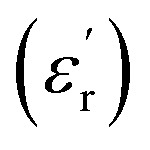
 and dielectric loss (tan *δ*), are illustrated in Fig. 3e and f. For pure epoxy, the dielectric permittivity hovers around 4.2, maintaining a nearly flat profile as the frequency increases up to 10 GHz. Nonetheless, the dielectric loss of the epoxy increases from 0.014 to 0.052 as increasing the frequency from 1 to 10 GHz, respectively. Epoxy-type dielectrics are widely used as low-permittivity substrate materials. However, due to their relatively high loss values (tan *δ* ≥ 0.02), their usage is limited where high speed and high operating frequency are required. Strategies to lower their intrinsic dielectric loss are mainly either modifying the molecular structure by reducing its polarity^2,35^ or incorporating low-loss fillers.^15^ As in the latter strategy, Alf is added to the epoxy matrix with varying wt% loading up to 20 wt% (20Alf). The permittivity of the composites shows a gradual increase with the addition of Alf up to 15Alf, and then upon the addition of 20Alf, a significant increase in the permittivity is observed (Fig. 3e) The dielectric loss of composites fluctuates up to 6 GHz but exhibits a downward trend and greater reduction in the dielectric loss upon filler loading, particularly between 6 and 10 GHz compared to that of pure epoxy (Fig. 3f).

In general, the metallic inclusions in a flake form significantly enhance the permittivity, which is due to effectively serving as conductive plates within the polymer matrix and forming numerous micro–nano capacitors and interfaces, thereby increasing the capacitive storage of the composites, followed by an increase in dielectric loss. The charges redistribute across the surface of the metallic fillers, forming electric dipoles that vary with frequency. As these mobile charges move through the material, energy dissipation occurs, contributing to the increased dielectric loss. In contrast, in our case, aluminum metal is coated by an insulating layer of stearic acid over its self-passivated oxide layer. This hybrid organic–inorganic coating can act as a barrier layer between the conductive Al metal core and epoxy host. This interlayer inhibits the charge accumulation and electron movement between neighboring Al flake particles, resulting in a weaker interfacial polarization,^59^ as long as the Alf loading is maintained below 15 wt%. Additionally, due to the hydrophobic tails of stearic acid, the flakes acquire a hydrophobic character (Fig. S12b and c†), which enhances their dispersion and helps maintain the distance between flakes within the epoxy matrix.^60^ This can effectively prevent agglomeration and close contact with neighboring flakes up to a certain loading level. In 20Alf, the abrupt increase in permittivity and a dielectric loss peak observed from 1 to 5 GHz can be associated with the increased number of interfaces created and reduced distance between the flakes and the insulating host. In this case, Maxwell–Wagner–Sillars interfacial polarization can likely take place at lower frequencies in which the movement of the charge carriers from the conductive fillers can cause a dipolar response from the insulating epoxy matrix and also the corresponding interface, leading to a slight dispersive dielectric behaviour in the permittivity and dielectric loss.^29,47,59^ Due to decreased distance between metallic flakes(clusters) upon increasing the Alf content within the epoxy host, the formed dipoles within the interface may interact with neighboring clusters and induce anomalous dielectric dispersion at low frequency, which is known as inter-cluster exchange proposed by Dissado and Hill.^61,62^ Particle aggregation and clusters can also lead to non-uniform electric field distribution and local energy dissipation within the composites. This effect is particularly evident in the 20 wt% Alf sample, where pronounced particle aggregation is observed (Fig. S11†), corresponding to the increased dielectric loss.

**Fig. 4:**
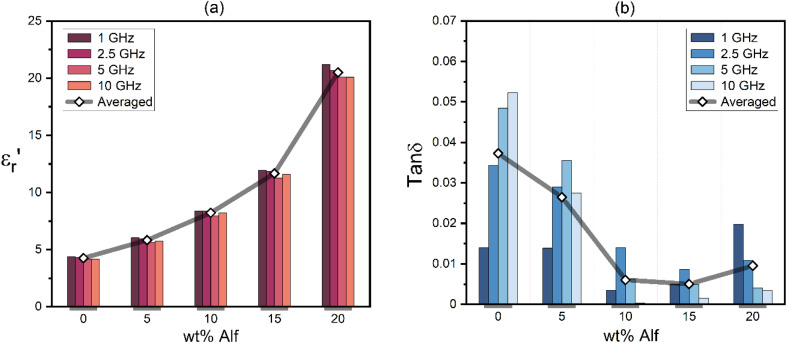
Dielectric properties of epoxy and Alf-added epoxy composites at selected frequencies; (a) the permittivity, 
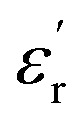
, and (b) dielectric loss, tan *δ*, with various wt% Alf. Averaged values are incorporated to depict the trend as a function of Alf loading.

The values of permittivity 
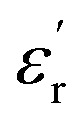
 and dielectric loss tan *δ* for pure epoxy and Alf-loaded composites at selected frequencies are demonstrated in Fig. 4. The values are averaged within the frequency and corresponding composition in order to depict the trend of the dielectric properties. The averaged permittivities of epoxy, 5Alf, 10Alf, 15Alf and 20Alf, are found to be 4.2, 5.8, 8.2, 11.6 and 20.5, respectively, and their corresponding dielectric losses are 0.037, 0.026, 0.006, 0.005 and 0.009. At the maximum loading of aluminum flakes (20Alf), the permittivity of the composite shows a notable increase, nearly 5 times over the selected frequencies. Simultaneously, the dielectric loss upon the filler loading shows a downward trend. The most prominent reduction in the loss is observed at higher frequencies (≥5 GHz). This observation holds significant relevance for devices operating within this frequency range.

In the case of low filler content (5Alf), the permittivity of the composite was similar to that of epoxy, yet the dielectric loss remained lower than that of epoxy. Hence, for the sake of comparison, 5 wt% Alf added epoxy resin is employed for glass fabric (GF) reinforced epoxy-based substrates in the subsequent section.

In order to verify the application potential, the composites were then fabricated in a laminate form, composed of a plain-woven glass fabric as reinforcement and epoxy resin mixture as a host with and without filler material, mimicking the FR4 layout as shown in Fig. 1, which is widely used for PCBs.

**Fig. 5:**
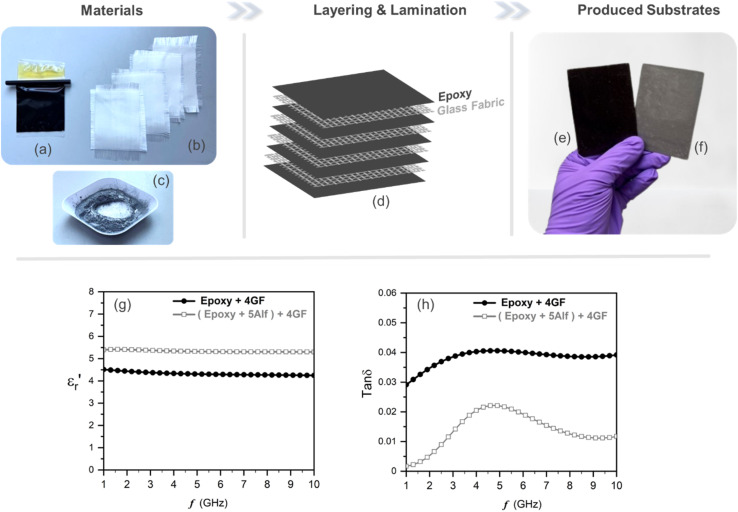
Fabrication of glass fabric reinforced epoxy substrates; materials include (a) epoxy resin mixture, (b) four layers of glass fabric and (c) aluminum flakes (Alf); (d) illustration of constructing layers; photographs of fabricated substrates (e) without Alf and (f) with Alf. Frequency-dependent dielectric properties of the fabricated glass fabric-reinforced epoxy-based substrates; (g) the real part of permittivity, 
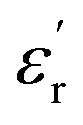
, and (h) dielectric loss, tan *δ*. Epoxy + 4GF refers to the substrate consisting of four layers of glass fabric embedded within epoxy resin, while (epoxy + 5Alf) + 4GF is the substrate comprising four layers of glass fabrics embedded within epoxy resin containing a 5 wt% Alf filler.

Materials used in fabricating glass fabric-reinforced epoxy-based substrates are shown in Fig. 5a–c. An equal amount of epoxy resin mixture, both with and without 5 wt% aluminum flake (Alf) filler, is cast onto a PTFE sheet and subsequently layered with four woven-glass fabric sheets, as illustrated in Fig. 5d. Following lamination and the curing process, substrates without and with Alf are obtained, as demonstrated in Fig. 5e and f, respectively.

Frequency-dependent dielectric properties of the fabricated substrates are given in Fig. 5g and h. The permittivities range from 4.5 to 4.3 for the substrate without 5Alf, and from 5.4 to 5.3 for the substrate with 5Alf added. On the other hand, the addition of 5Alf reduces the dielectric loss of the substrate without 5Alf, decreasing it from 0.029 to 0.001 at 1 GHz, from 0.041 to 0.022 at 5 GHz, and from 0.039 to 0.011 at 10 GHz. The observed decrease in dielectric loss when using Alf in an epoxy medium aligns well with the results obtained from Alf-added epoxy composites illustrated in Fig. 4b.

Most electronics work with alternating current, AC. Therefore, dielectric properties of a material can become time- and, therefore, frequency-dependent. How a material responds to the speed of an electric field's oscillation determines its frequency response in stored energy and energy loss, which are associated with *ε*_r_ (*ω*) and tan *δ* (*ω*), respectively. The amount of dielectric energy loss *W* can be calculated by the following equation:^15^

1 *W* =*Kω*^2^Δ(*ε*′ tan *δ*)

where *K* is a constant, *ω* is the signal frequency, *Δ*^2^ is the potential gradient, *ε*′ is the real part of complex permittivity, and tan *δ* is the dielectric loss tangent. Tan *δ* is the ratio of the imaginary part (*ε*′′) to the real part (*ε*′) of complex permittivity, and thus the part (*ε*′ tan *δ*) in the equation yields the imaginary part (*ε*′′), also known as the loss factor, which shows direct relation to the energy loss in a dielectric.

Another aspect regarding the significance of dielectric loss in high-frequency transmission substrate and microwave electronic packaging applications is the transmission speed and signal integrity. The amount of signal energy utilized in most microelectronics is relatively small, and there is a prevailing industry trend towards lower power inputs. However, loss mechanisms within the materials comprising the circuitry absorb some of this energy, leading to the degradation of signal integrity. The relationship among signal transmission speed, attenuation, relative permittivity and dissipation factor can be depicted as following expressions:^1,6,36^2
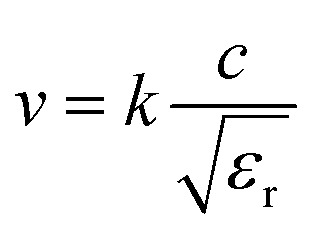
3
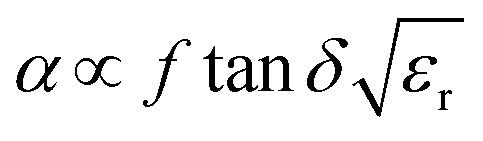
where *v* and *α* are signal transmission speed and attenuation, respectively, *k* is the coefficient, *c* is the speed of light, *ε*_r_ is relative permittivity and *f* is the frequency. From this perspective, it can be seen that maintaining low permittivity and reducing dielectric loss tangent of a dielectric substrate material can potentially improve transmission speed and reduce attenuation of signal transmission in high frequency.

Losses also generate heat within the device, leading to performance degradation and needs to be managed or eliminated, using high thermal conductive copper cladding, for example.^15^ Heat removal from electronic circuits also tends to increase cost. Therefore, the dielectric loss of a PCB is a key criterion in designing the microwave circuitry in today's wireless devices. Although the developed filler is an insulating coated aluminum metal flake, based on the properties of the materials, effective thermal conductivity of the composite could increase as aluminum (234 W m^−1^ K^−1^) has a much higher thermal conductivity than the epoxy resin matrix (0.17–0.21 W m^−1^ K^−1^).^63^ As a result, when embedded in the epoxy host, this coated-metal filler not only provides low dielectric loss characteristics but also potentially could enhance the thermal capabilities of PCBs, which need to be verified in further work. This electrical and thermal dual functionality are vital parameters in current substrate materials, and paves the way for innovative filler materials for future dielectric substrates.^42^ Moreover, the ability to adjust permittivity and loss values by incorporating Alf particles into polymer hosts makes them highly suitable to be used for the development of miniaturized devices with low power consumption, and integration into wearable electronics, as well as emerging technologies such as devices facilitating interaction between humans and computers, and smart sensing devices.^36^

## 3. Conclusions

Stearic acid-coated aluminum flakes are designed as a low-loss filler to be used in epoxy-based dielectric substrates. A simple and effective approach for fabricating such metallic filler with protective layers is demonstrated and their impact on the dielectric properties of an epoxy matrix is investigated. It is found that the incorporation of aluminum flakes into an epoxy matrix is a highly effective means of reducing the dielectric loss tangent (tan *δ* or *D*_f_) of the epoxy. This incorporation can decrease the dielectric loss of the host material by an order of magnitude. To explore the application space for this material, this method is subsequently applied to glass fabric-reinforced epoxy-based substrates, by mimicking the internal structure of FR4 substrates, which are widely used in printed circuit boards. The dielectric properties of fabricated substrates show minimal variation in the permittivity 4.5 and 5.4, whereas significant reductions in dielectric loss from 1 to 10 GHz are observed when aluminum flakes are added as a filler into the epoxy resin mixture. The results show that surface-modified aluminum flakes can be a great potential filler material for high-frequency and high-speed substrate applications that require low loss.

## 4. Experimental section

Irregularly shaped aluminum powder with an average particle size ranging from 7 to 15 μm was obtained from Alfa Aesar. Stearic acid was obtained from Fisher Scientific Ltd. A high-energy planetary ball mill (Fritsch, Pulverisette 6) equipped with a stainless-steel milling bowl and stainless-steel milling balls was utilized to produce flake-shaped metal particles. For every 4 g of as-received aluminum powder, 1 gram of stearic acid and 15 mL of toluene were added. The milling process was conducted in an 80 mL stainless steel bowl containing 150 g of 3 mm stainless steel milling balls. The milling procedure lasted for 4 hours, with a milling speed of 300 rpm and 24 cycles consisting of 10 minutes of effective milling followed by 5 minutes of pause for cool-down. Subsequently, the resulting flaked metal particles (Alf) were dried overnight in a 60 °C vacuum oven. The described processing method was found to be optimal for achieving effective flake formation and enhanced dielectric performance. Additional details on filler optimization are provided in the ESI,† including investigations of various milling times (Fig. S7 and S8†), optimising amounts of stearic acid (Fig. S1 and S2†), and alternative organic additives such as behenic acid, lauric acid, perfluorodecanoic acid, and propyltrimethoxysilane (Fig. S3–S6†). As a control experiment, Al flakes were also produced without the addition of stearic acid (Fig. S12†). For safety reasons, this process was conducted in a polypropylene jar filled with nitrogen without any solvent using a low-energy benchtop roller. Milling was performed for up to 192 hours, with the size of the Al flakes regularly monitored until they were comparable to those produced using stearic acid.

The epoxy resin utilized throughout this study is a commercial product, ER1448, purchased from Electrolube, UK. This resin comprises two components: the black part consists of the resin (with a density of 1.83 g ml^−1^), while the amber part contains the hardener (with a density of 1.02 g ml^−1^). The supplier specifies a mix ratio of 5.53 : 1 (resin to hardener) in volume. The epoxy composites containing Alf were prepared using the following experimental procedures: first, a certain wt% of Alf was mixed into the resin solution and sonicated for 30 minutes to achieve a homogeneously dispersed suspension, free from visible agglomerations and residual fillers along the edges of the beaker. Subsequently, the hardener was added to the suspension of fillers and vigorously mixed for 5 minutes. The resulting suspensions were then poured into silicone molds and transferred to a vacuum oven set at 60 °C where they were cured for 2 hours. Following curing, the composites were removed from the oven and kept at room temperature to ensure a complete curing process for at least 48 hours before dielectric measurements were conducted. In this work, aluminum flakes loading 5, 10, 15, and 20 wt% into the epoxy matrix are denoted as 5Alf, 10Alf, 15Alf, and 20Alf, respectively.

Plain weave woven glass fabric (GF) was obtained from Ybaymy, UK, and cut into square pieces measuring 100 mm. A 5 wt% Alf-added epoxy resin mixture, prepared as previously described, was used for layering. One gram of resin mixture was applied onto a PTFE sheet using a spatula, followed by a layer of glass fabric. This was then compacted using a stainless-steel roller by hand, and another gram of resin mixture was applied onto the glass fabric. This process was repeated for all four layers of glass fabric. Finally, the top layer was covered with 1 gram of resin mixture and a PTFE sheet was covered on the top layer. The assembly was left to settle for 30 minutes before undergoing hot-press lamination using heated stainless-steel plates at 60 °C for 5 minutes under 1 ton of pressure. The laminated substrates were then transferred to a vacuum oven set at 60 °C and cured for 2 hours. After curing, the substrates were removed from the oven and kept at room temperature for at least 48 hours before dielectric measurements were conducted. The substrates were then cut into dimensions of 85 mm × 60 mm × 1 mm (length × width × thickness).

Scanning electron microscopy (SEM) images of the Al powder, flakes and epoxy/Alf composites were acquired by Hitachi S-4800 equipped with an Oxford INCA energy-dispersive X-ray spectroscopy (EDS) detector which is used for elemental mapping. X-ray diffraction (XRD) results were obtained using a Bruker D8 Discover X-ray diffractometer with Cu-Kα radiation. The samples were scanned in the 2*θ* range of 5° to 80° with a step interval of 0.01314°.

For dielectric measurements, the dielectric assessment kit for thin layers (DAK-TL2) from SPEAG (Schmid & Partner Engineering AG, Switzerland), which is based on the open coaxial probe method, was used to perform high-frequency dielectric spectroscopy. The DAK3.5-TL2 probe was used in combination with a ZVL (Rohde & Schwarz, Munich, Germany) to perform measurements over the microwave frequency range. The measurement resolutions are set to 100 MHz covering the range from 1 GHz to 10 GHz. The DAK-TL2 system was calibrated using the standard 3-point calibration prior to each measurement session: open-circuit, short-circuit (copper strip), and de-ionized water and/or PTFE as the load. A force of 800 N was applied during the short calibration to ensure a good contact between the probe and the copper strip. For liquid composites, dielectric properties were measured using an Agilent N9917A FieldFox Microwave Network Analyzer and a Keysight 85070E Dielectric Probe Kit. The instrument's measurement frequency range was set from 1 to 10 GHz with 1001 data points. A three-point calibration method—comprising open-circuit, short-circuit, and distilled water measurements—was employed. All measurements were conducted at room temperature. Liquid and liquid composite samples were mixed and held in borosilicate glass vials with PTFE-lined caps. The probe was immersed 10 mm below the surface of the liquid samples. The relative complex permittivities, including their real and imaginary components, were recorded accordingly. Calibration and measurement steps were repeated multiple times to ensure data repeatability. Additionally, compounds with well-characterized relative complex permittivity spectra from the literature were measured after each calibration to validate the reliability of the results.

## Data availability

The data that support the findings of this paper are available from the corresponding authors, Prof. Jianliang Xiao and Prof. Yi Huang, upon reasonable request.

## Conflicts of interest

There are no conflicts to declare.

## Supplementary Material

RA-015-D4RA07419J-s001
