# Supplementary material for: Designing a filler material to reduce dielectric loss in epoxy-based substrates for high-frequency applications
Source: RSC Adv. 2025 Jan 9;15(2):754–63. doi: 10.1039/d4ra07419j (PMC11714422; doi:10.1039/d4ra07419j)
Supplement: RA-015-D4RA07419J-s001 [file RA-015-D4RA07419J-s001.pdf]

Supplementary Information (SI) for RSC Advances.
This journal is © The Royal Society of Chemistry 2024

Designing a Filler Material to Reduce Dielectric Loss in Epoxy-Based Substrates for High-Frequency Applications

Ilkan Calisir,[a] Elliot L. Bennett,[a] Xiantao Yang,[b] Jianliang Xiao[a†] and Yi Huang[b†]

[a] *Department of Chemistry, University of Liverpool, Crown Street, Liverpool, L69 7ZD, United Kingdom*
[b]*Department of Electrical Engineering and Electronics, University of Liverpool, Brownlow Hill, Liverpool, L69 3GJ, United Kingdom*

†*Corresponding authors:*
Professor Yi Huang huangyi@liverpool.ac.uk;
Professor Jianliang Xiao jxiao@liverpool.ac.uk

1. Optimisation, Screening and Evaluation of Al flakes/powders

The following sections detail the optimization and screening processes for Al flakes, as well as the evaluation of the resulting flakes through dielectric data and microstructural analysis. Polyethylene glycol 600 (PEG600) was used as the liquid matrix to more effectively benchmark the modified Al flakes in terms of dispersibility and dielectric loss.

1.1 Optimisation of Al powder-to-stearic acid ratio:

Optimisation was performed based on the weight ratio of Al to stearic acid, as follows; for each 4 g of irregularly shaped aluminum powder, 0.1, 0.5, 1.0 and 1.5 g of stearic acid and 15 mL of toluene were added. Milling was performed in an 80 mL bowl with 150 g of 3 mm steel balls at 300 rpm for 4 hours, using 24 cycles of 10-minute milling and 5-minute cooling. The resulting flakes (Alf) were dried overnight in a 60°C vacuum oven. Dielectric properties of the resultant flakes with varying ratios were evaluated in a liquid matrix, PEG600, as shown in Fig. S1. Excess stearic acid on the flakes was observed at a 4:1.5 ratio, as shown in Fig. S2.

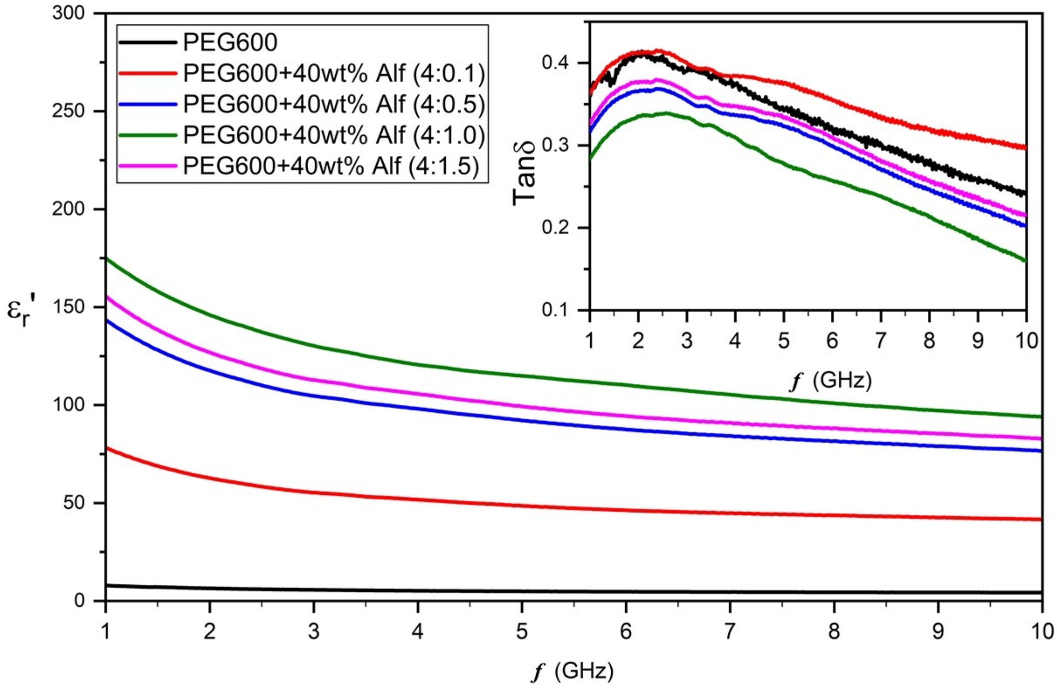

Fig. S1. Comparison of the frequency-dependent dielectric properties of polyethylene glycol 600 (PEG600) and composites containing 40 wt% Al flakes (Alf). The Alf was prepared via 4h-ball milling with varying aluminum powder-to-stearic acid weight ratios, labeled as 4:0.1, 4:0.5, 4:1.0, and 4:1.5.

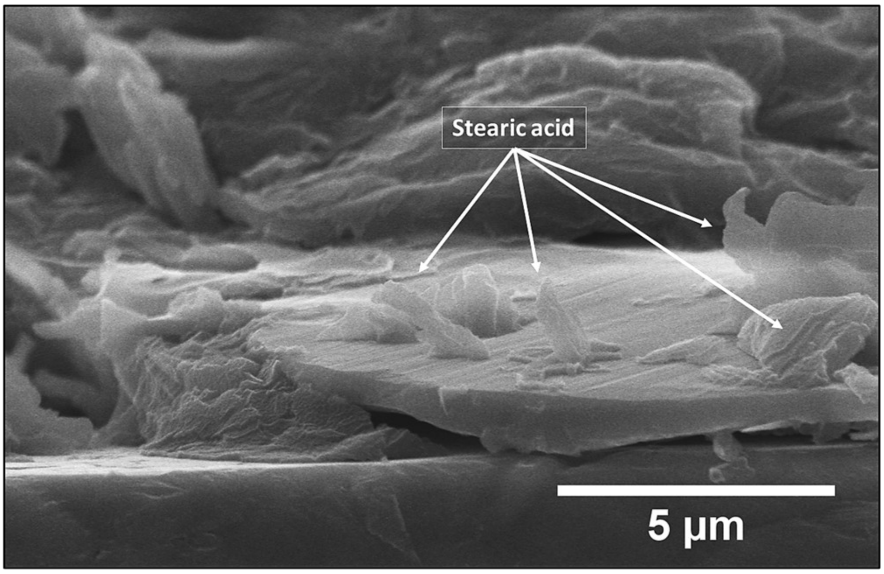

Fig. S2. SEM images of stearic acid-coated Al flakes with a 4:1.5 Al-to-stearic acid weight ratio.

1.2 Organic coating screening on Al flakes

Stearic acid, behenic acid, lauric acid, and perfluorodecanoic acid were screened and utilized as organic coatings on Al particles. Their chemical structures are illustrated in Fig. S3, and the experimental method is provided below.

- *Stearic acid (SA) on Al:* For each 4 g of irregularly shaped aluminum powder, 1 g of stearic acid and 15 mL of toluene were added. Milling was performed in an 80 mL bowl with 150 g of 3 mm steel balls at 300 rpm for 4 hours, using 24 cycles of 10-minute milling and 5-minute cooling. The resulting flakes (Alf) were dried overnight in a 60°C vacuum oven.
- *Behenic acid (BA) on Al:* It was prepared using the same experimental procedure as described for 'Stearic acid on Al'
- *Lauric acid (LA) on Al:* It was prepared using the same experimental procedure as described for 'Stearic acid on Al'
- *Perfluorodecanoic acid (PFDA):* It was prepared using the same experimental procedure as described for 'Stearic acid on Al'

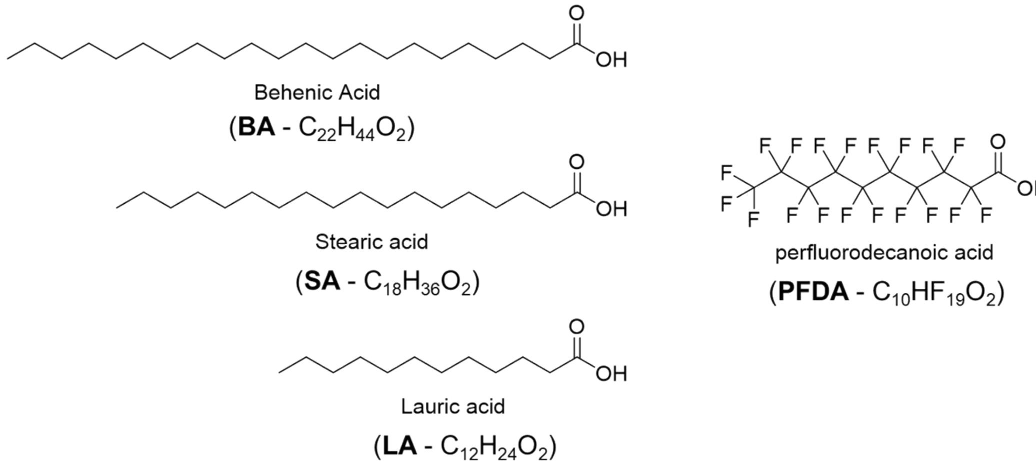

Fig. S3. 2D chemical structure of organic coatings used on Al flakes; behenic acid (BA), stearic acid (SA), lauric acid (LA) and perfluorodecanoic acid (PFDA)

In addition to screening SA, BA, LA, and PFDA coatings on Al flakes, we also evaluated the feasibility of coating Al flakes with propyltrimethoxysilane. This coating aims to improve dispersion and act as a third insulating layer on the flakes, as detailed in the experimental procedure below.

- *Propyltrimethoxysilane (PTMS) on Alf*: Stearic acid coated-aluminum flakes (10 g) were functionalized with 5 wt% PTMS in toluene (50 mL) under reflux at 125°C for 20 hours. The mixture was vacuum-dried, washed with ethanol, and centrifuged to remove residues. The flakes were then vacuum-dried for 4 hours, yielding free-flowing aluminum flakes with 5 wt% PTMS.

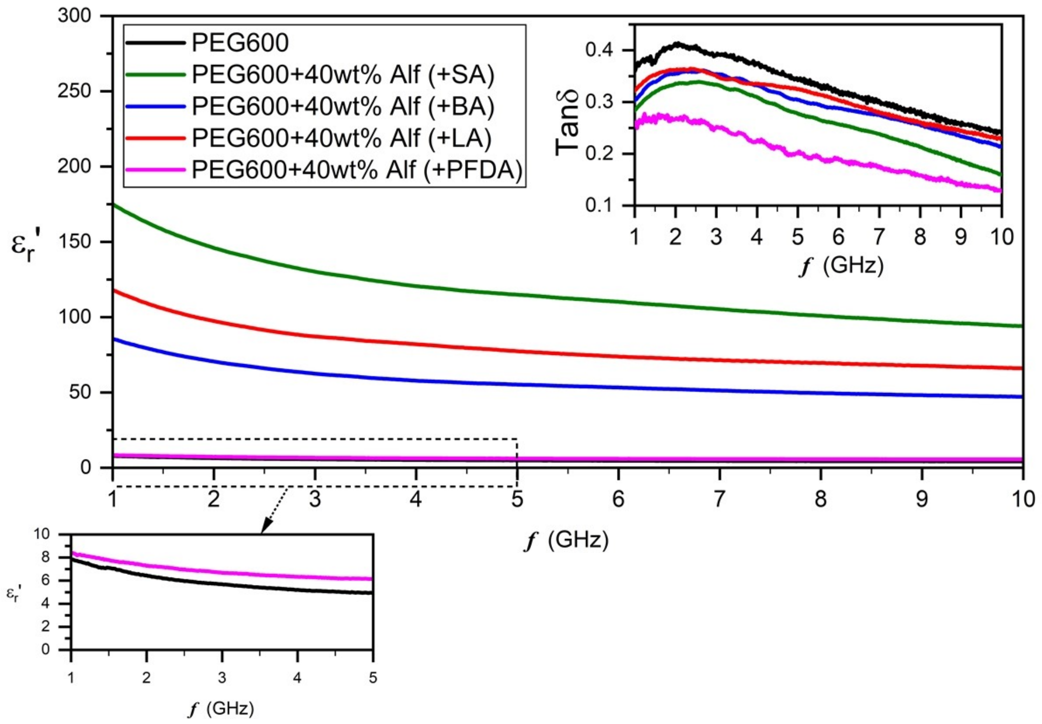

Fig. S4. Comparison of the frequency-dependent dielectric properties of polyethylene glycol 600 (PEG600) and composites containing 40 wt% Al flakes prepared with various organic additives. The Al flakes (Alf) were produced via ball milling with a 4:1 Al-to-additive weight ratio, followed by the same milling procedure described in the experimental section. The organic additives used were stearic acid (SA), behenic acid (BA), lauric acid (LA), and perfluorodecanoic acid (PFDA).

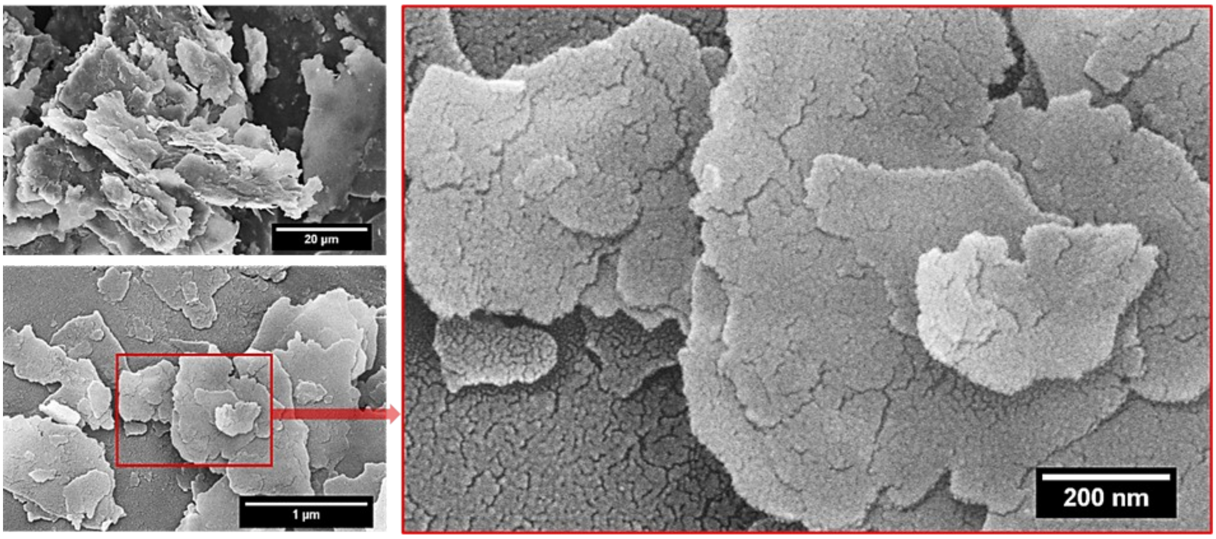

Fig. S5. SEM images of aluminum flakes prepared with perfluorodecanoic acid (PFDA) after 4 hours of milling, shown at various magnifications. Severe fragmentation of the flakes is observed, with particles progressively transforming into fine, nano-sized particles. This indicates poor adhesion of PFDA on the Al surface which is likely due to the perfluoro-chain of PFDA and this led to severe oxidation of Al flake surfaces during ball milling.

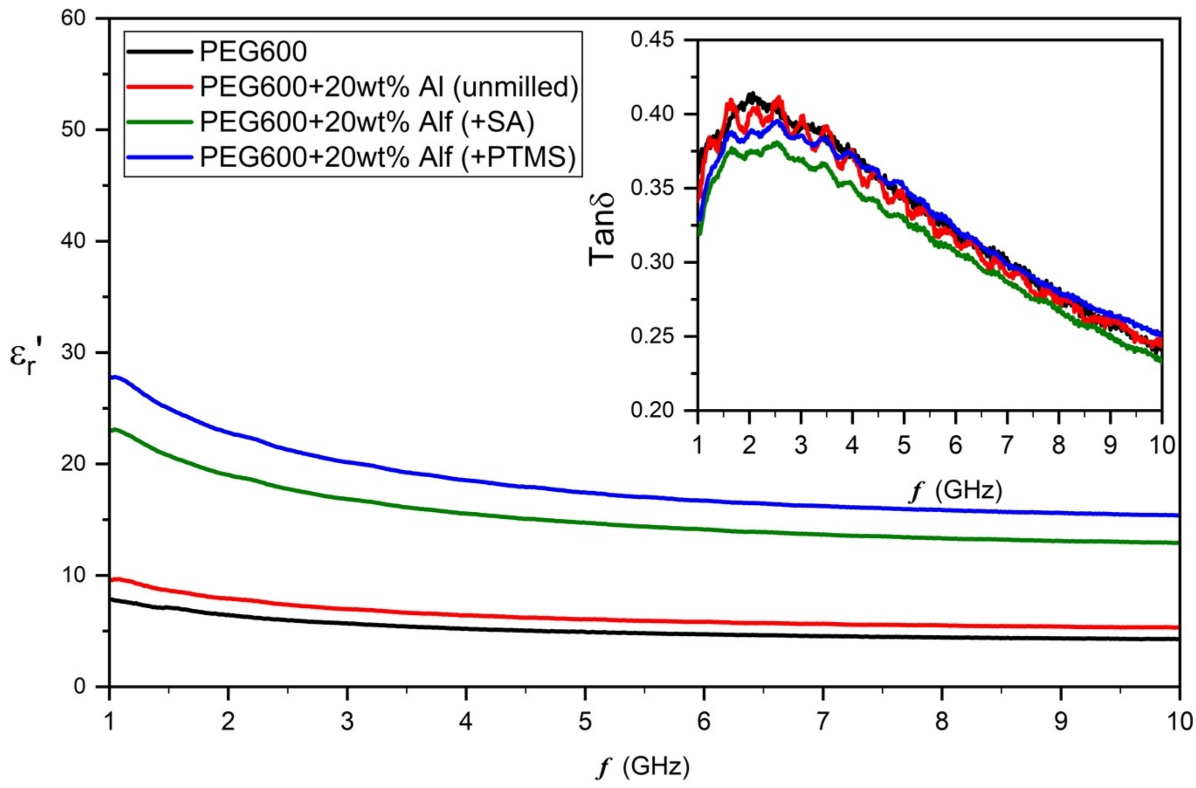

Fig. S6. Comparison of frequency-dependent dielectric properties of polyethylene glycol 600 (PEG600), unmilled Al powder, Alf (stearic acid added, SA) and Alf (propyltrimethoxysilane added, PTMS) composites.

1.3 Optimisation of milling time:
High-energy ball-milling on irregular shaped Al particles was performed up to 10 hours for Al flake formation, collecting data at 1-hour, 2-hour, 4-hour, 6-hour, and 10-hour intervals. This procedure resulted in varying lateral sizes and thicknesses of Al flakes, as shown in Fig. S7. The size and thickness of the Al flakes decreased with increasing milling time. The correlation between milling time and dielectric properties is provided in Fig. S8. The optimum dielectric performance was achieved with a 4-hour milling time. Milling beyond 4 hours resulted in flakes prone to agglomeration due to their reduced size, leading to decreased dielectric permittivity and a slight increase in dielectric loss when incorporated into PEG600, compared to the 4-hour milled Al flakes.

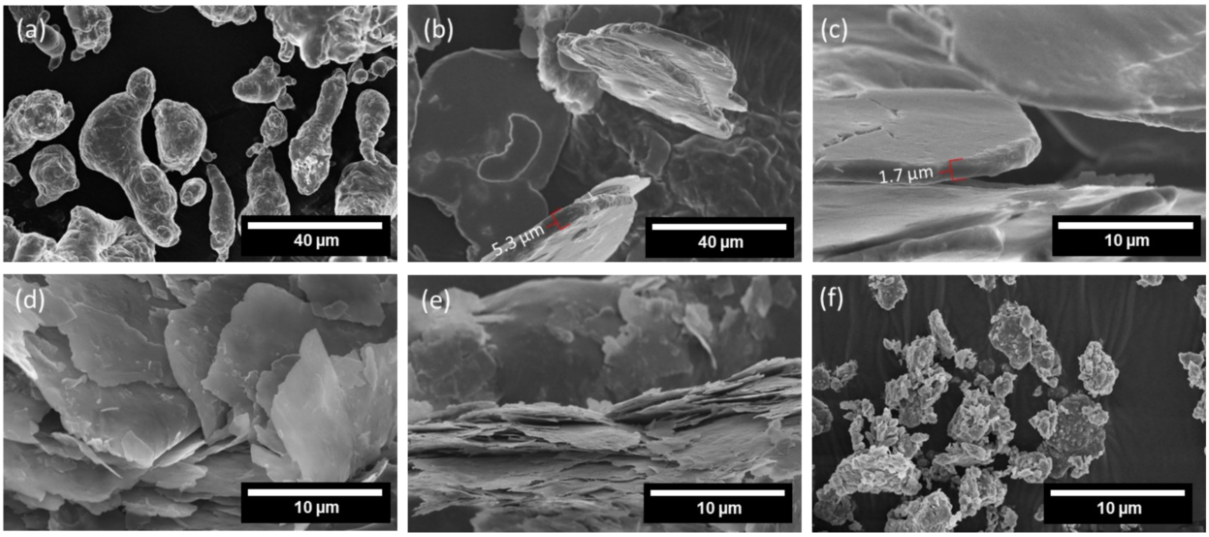

Fig. S7. SEM images of stearic acid-coated Al particles at different milling stages: a) unmilled (as-received), b) 1-hour milled, c) 2-hour milled, d) 4-hour milled, e) 6-hour milled, and f) 10-hour milled.

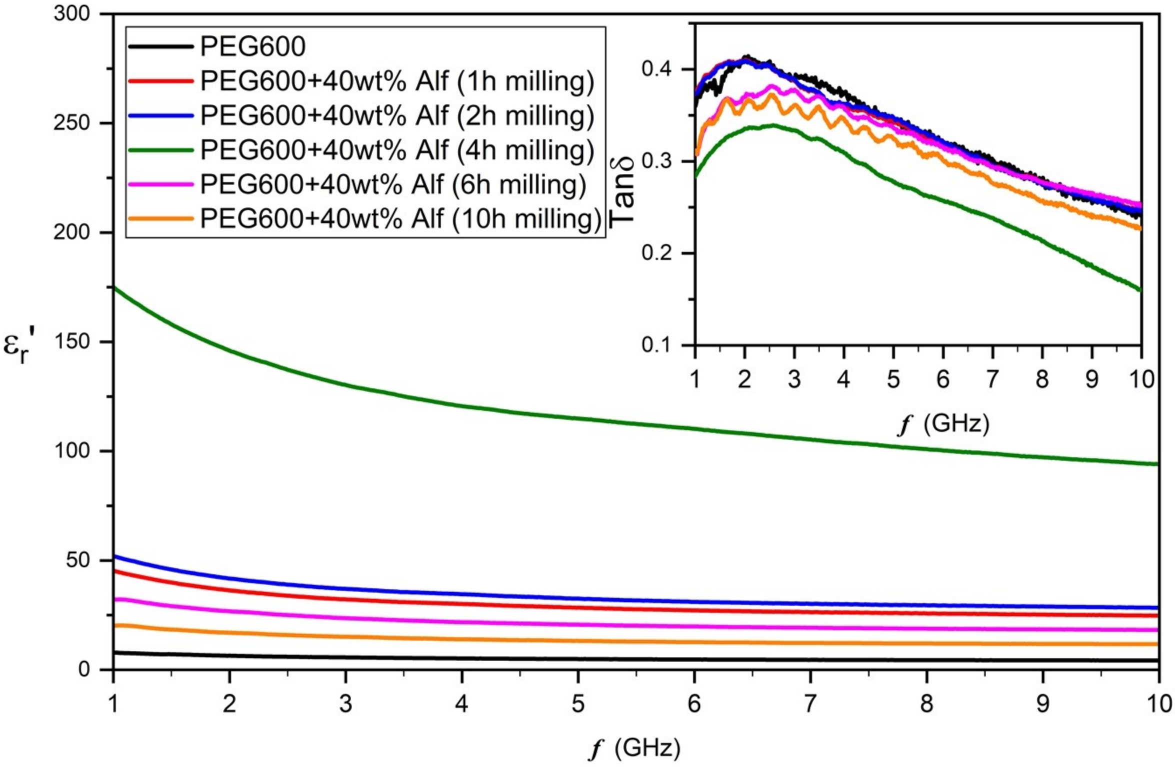

Fig. S8. Comparison of the frequency-dependent dielectric properties of polyethylene glycol 600 (PEG600) and composites containing 40 wt% aluminum (Al), which is prepared via ball-milling with varying milling times, up to 10 hours.

2. Microstructures of unmilled, milled Al particles and varying wt% Al-added epoxy composites

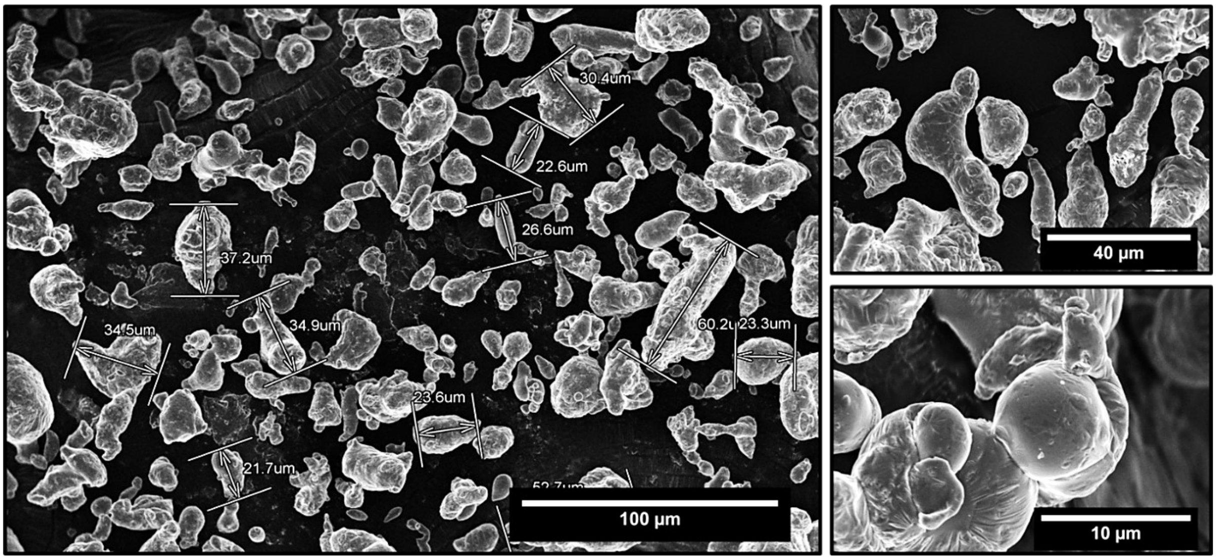

Fig. S9. SEM images of unmilled, as-received aluminum (Al) powders at various magnifications.

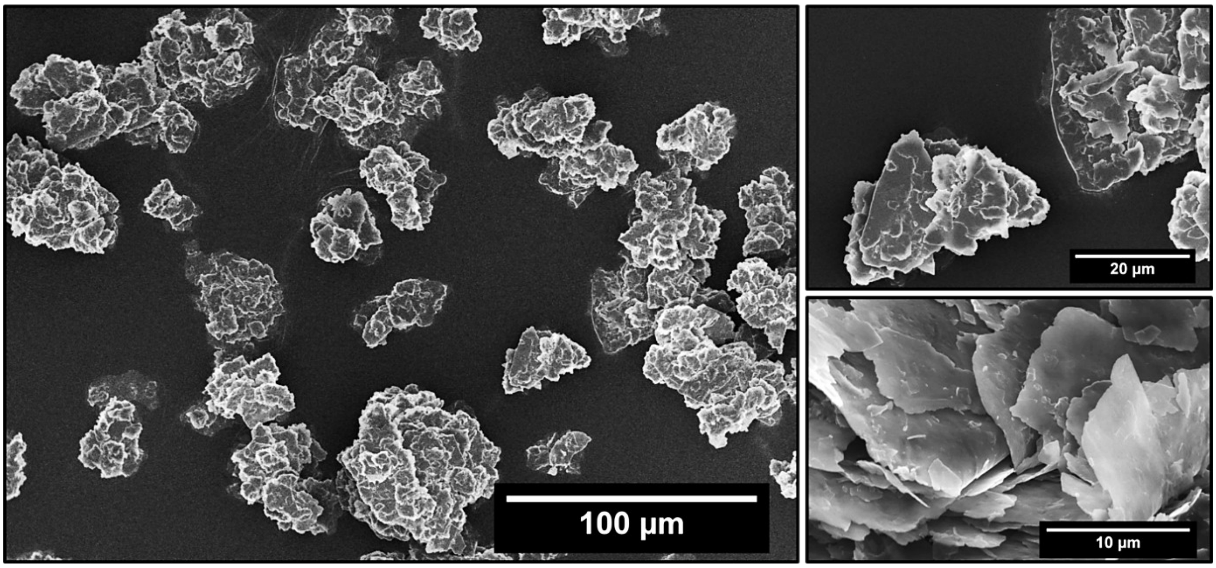

Fig. S10. SEM images of 4 h-milled stearic acid coated-aluminum flakes (Alf) at various magnifications.

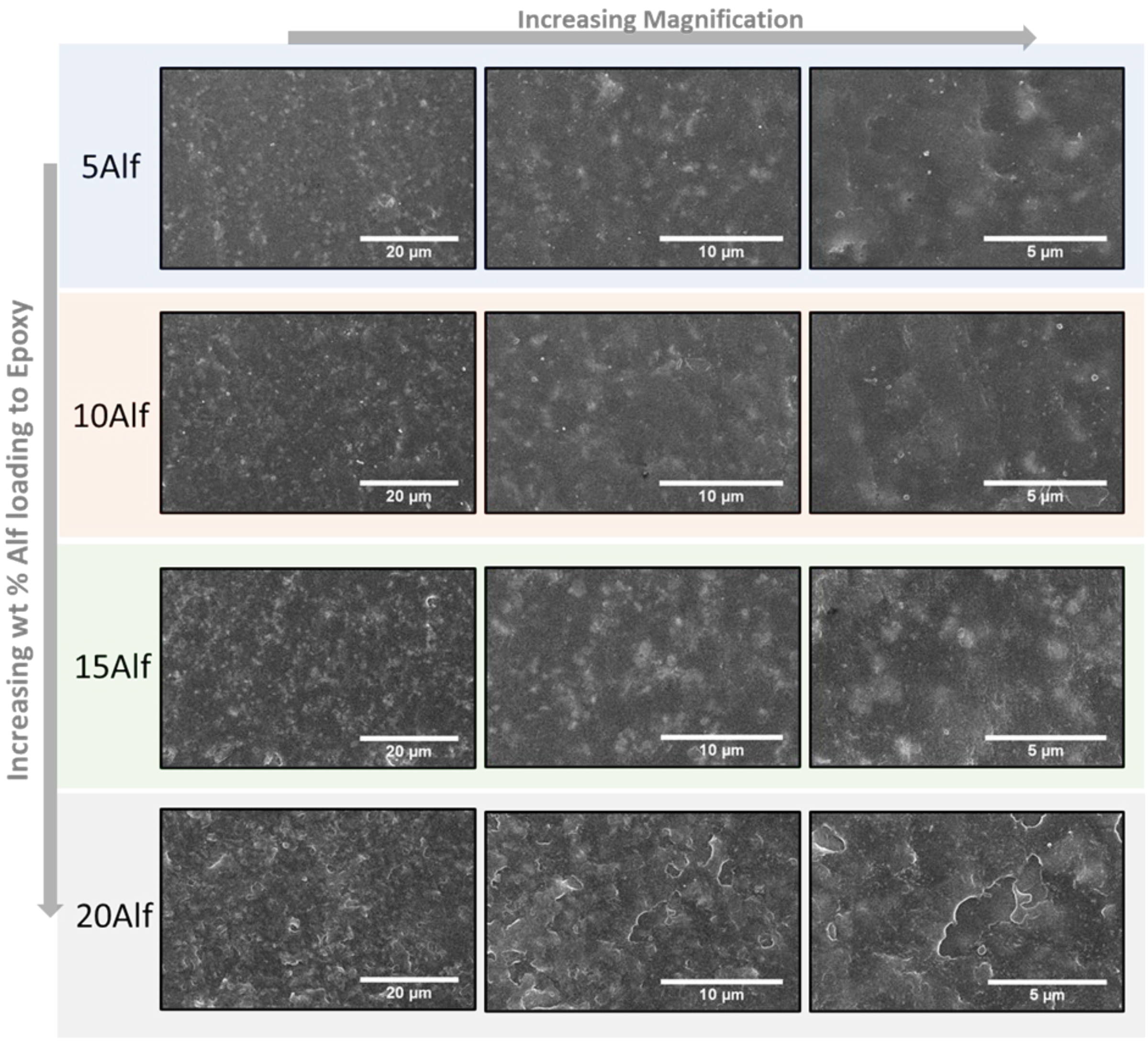

Fig. S11. SEM images of the surface of Alf-added epoxy composites at various magnifications, with varying wt% of Alf; 5, 10, 15 and 20, labelled as 5Alf, 10Alf, 15Alf and 20Alf, respectively.

3. Physical and dielectric characteristics of Al flakes milled with and without stearic acid

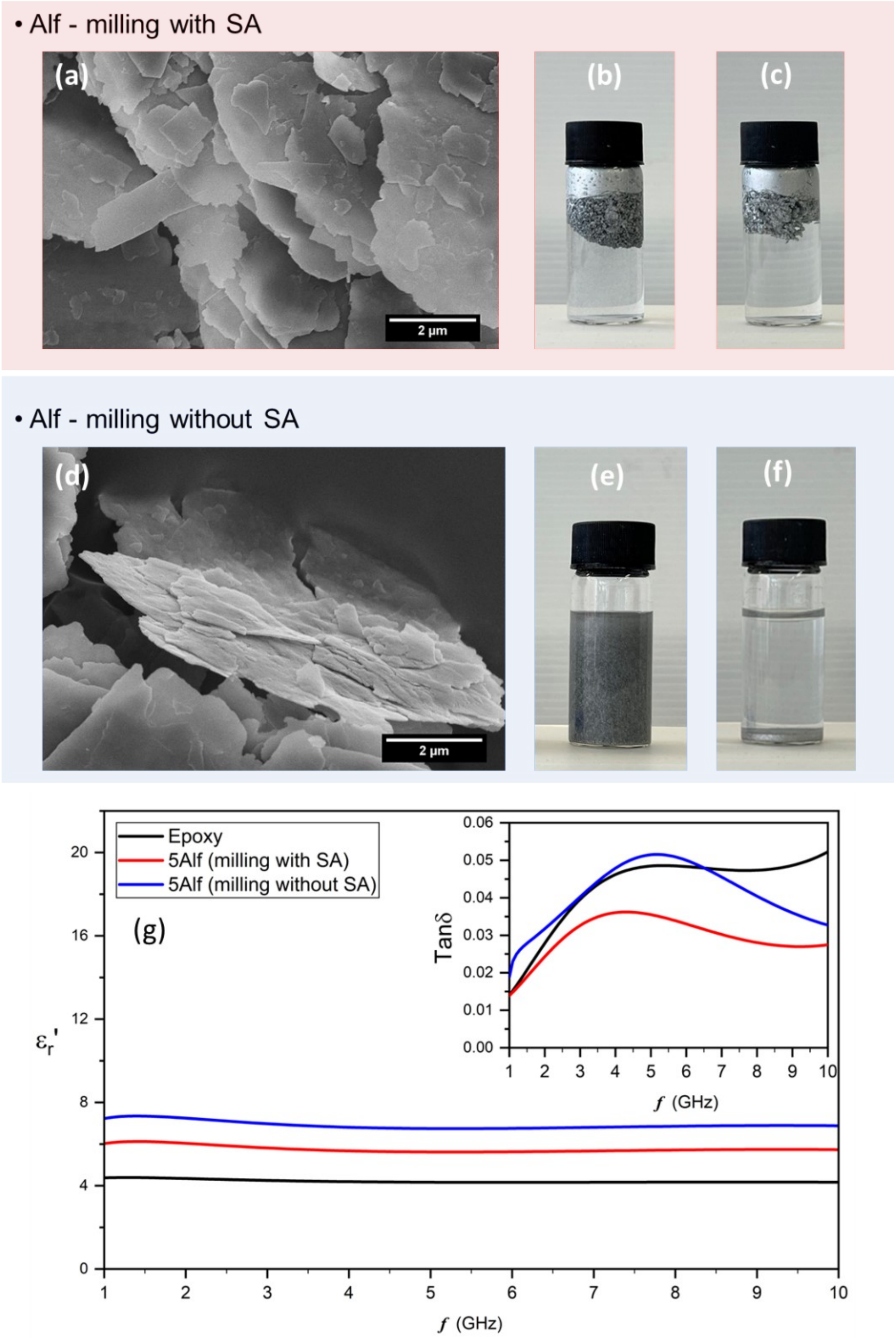

Fig. S12. Characteristics of Al flakes obtained through milling with and without stearic acid (SA): a,d) SEM images, b,e) vigorous mixing with distilled water in a vial, and c,f) after being left undisturbed for 24 hours at room temperature, demonstrating their hydrophobic nature. g) Comparison of the frequency-dependent dielectric properties of epoxy and its composite containing 5 wt% Al flakes (Alf), which were prepared via ball-milling with and without SA.
